# Calculation of active earth pressure on external corners with equal length on both sides in excavation engineering

**DOI:** 10.1038/s41598-023-29873-6

**Published:** 2023-02-16

**Authors:** Zhao Long, Hao Zeng, Shuaihua Ye, Weili Li

**Affiliations:** 1 Gansu CSCEC Municipal Engineering Investigation and Design Institute Co., Ltd., Lanzhou, 730000 China; 2 Key Laboratory of Disaster Mitigation in Civil Engineering of Gansu Province, Lanzhou University of Technology, Lanzhou, 730050 China; 3 Western Center of Disaster Mitigation in Civil Engineering, Ministry of Education, Lanzhou University of Technology, Lanzhou, 730050 China

**Keywords:** Civil engineering, Engineering

## Abstract

Taking the external corner with equal lengths as the research object, two failure modes of the equilateral external corner are established, and the active earth pressure calculation formula of the equilateral external corner in the limit state is further deduced when the external angle is 90°. The comparison between the theoretical calculation results and Midas/GTS simulation results shows that when the ratio of the side length *B* to the depth *H* is large, the sliding wedge failure will occur at the external corner, and the active earth pressure of the external corner in the range of wedge-shaped slider will not change with the change of size *B*. When the ratio of the side length *B* to the depth H is small, the displaced soil is composed of two parts, and the earth pressure varies with the change of size *B*. In this paper, the magnitude and distribution law of the active earth pressure obtained by the horizontal micro-layered limit equilibrium analysis method is similar to the three-dimensional simulation value of the Midas/GTS software, which can prove the feasibility and rationality of the theoretical calculation.

## Introduction

**Figure 1:**
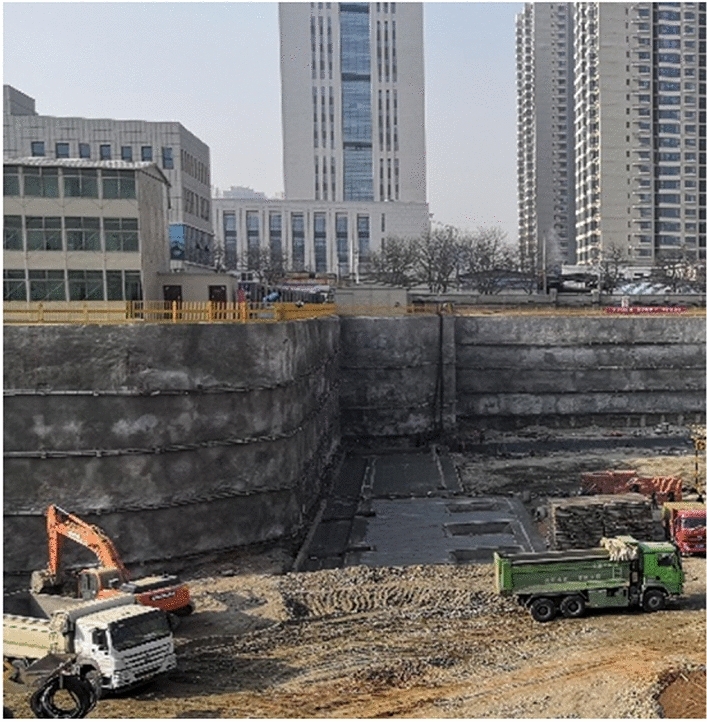
Picture of the external corner.

The construction site of excavation engineering is easily affected by surrounding buildings and all kinds of pipe networks, subways and comprehensive pipe corridors^1^. And the plane shape of the foundation pit becomes more irregular, leading to a large number of excavations with external corners. Due to the free surface on both sides of the external corner in the foundation pit, the stress distribution at the external corner is complex, and it is more sensitive to the surface stress level, the change in groundwater level and the stiffness of the support system. Therefore, special attention needs to be paid to the external corner, and it is necessary to do more research on the three-dimensional failure form of the external corner and the calculation of earth pressure in excavation engineering. The picture of the external corner in the excavation is shown in Fig. 1.

The research on the three-dimensional failure mode of the foundation pit is mostly aimed at the foundation pit with the regular plane shape (without the external corner), but the research on the three-dimensional failure mode of the external corner is less. In the three-dimensional study of the regular foundation pit, Ou^2–5^ present the concept of plane strain ratio PSR (the ratio of the maximum displacement at the midpoint of the pit wall in the three-dimensional model to the maximum displacement in the two-dimensional plane strain model) to describe the influence degree of the pit corner effect; and carried out a large number of studies on the pit corner effect. Based on the concept of PSR, through the numerical simulation of a large number of regular deep foundation pits, an empirical formula of PSR has been summarized and put forward by Finno^6^. Many scholars have also conducted monitoring tests and three-dimensional numerical simulations of the regular foundation pit^7–13^. Li et al.^14^ believe that the three-dimensional failure mechanism is related to the soil arching effect and then deduce the three-dimensional earth pressure calculation equation for internal corners. Based on this theory, Lin et al.^15^ simplified the curve of the soil arching and then obtained the equations to calculate the three-dimensional active earth pressure of the retaining wall using the horizontal micro-layered limit equilibrium analysis method^16^. In addition, many scholars^17–20^ have studied the three-dimensional earth pressure calculation of internal corners according to different conditions. These research results show that the foundation pit has obvious three-dimensional characteristics, and it is essential to use a three-dimensional model to calculate the earth pressure in the foundation pit. However, all the above studies take the internal corner as the research object, and previous studies^21–24^ have shown that the external corner is not conducive to the safety and stability of the foundation pit, so it is necessary to pay more attention to the external corner. Zhang^22^ study found that when the external corner displacement occurs, the soil around the positive angle will produce deformation similar to the wedge-shaped slider. Pan et al.^25^ pointed out that when the side length of the external corner is identical to the excavation depth, the slip surface of the external corner is approximately spherical. Wang et al.^26^ classified the external corner according to the proportional relationship between the length of both sides for the external corner and the plane size of the foundation pit, and the research shows that the side length of the external corner is an essential factor affecting the deformation. Moradi^23^ studied the effect of the external corner on the deformation of the soil-nailed wall through experiments and evaluated the influence of the nail arrangement on the performance of the soil-nailed wall. Wu et al.^27^ studied the spatial effect of the soil-nailed wall by using the three-dimensional elastic–plastic model, and the results show that the internal corner is beneficial to the stability of the foundation pit, while the external corner is very disadvantageous to the stability of the foundation pit. Research at this stage shows that the external corners protruding from the foundation pit are more prone to excessive deformation, and are more likely to be unstable or damaged. And the earth pressure is closely related to the deformation, so it is necessary to study the earth pressure of the external corner.

In the past, most of the studies on the pit corner effect of the foundation pit are based on three-dimensional numerical simulation. Compared with the internal corner of the foundation pit, research on the external corner is very rare, and there is still a lack of theoretical research on the external corner of the foundation pit. Therefore, this paper studies the three-dimensional failure mode of the external corner in the foundation pit and puts forward two different failure models related to the side length of the external corner. The horizontal stratification analysis method is used to theoretically analyze the external corner with a turning angle of 90°, and the corresponding earth pressure calculation formula is obtained. The three-dimensional finite element numerical calculation is carried out using Midas/GTS software, and the simulation results are compared with the theoretical method. It is verified that the two failure models proposed in this paper are aimed at the external corner with the same length on both sides, and the theoretical calculation results of earth pressure are consistent with the simulation results of the software.

### Calculation of active earth pressure

#### Failure mode analysis of the external corner with equal length on both sides

**Figure 2:**
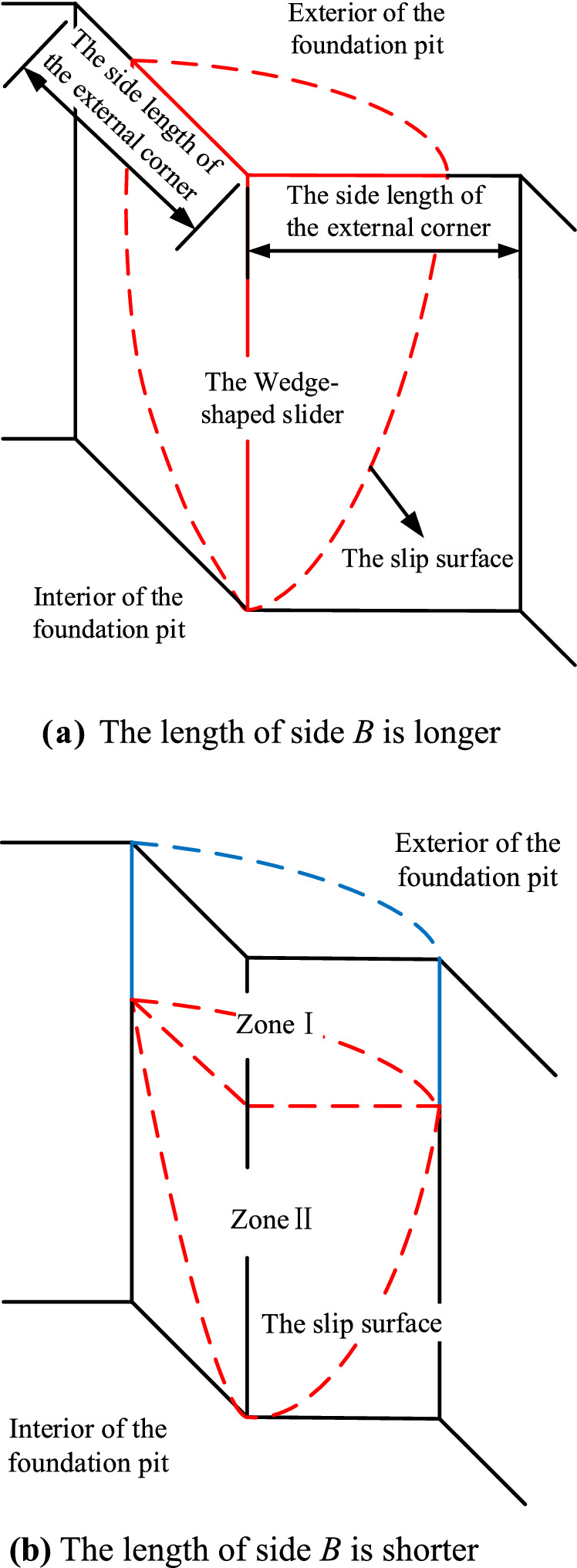
The failure mode of the external corner with equal lengths on both sides.

Two different failure modes are proposed for the external corner with equal length on both sides. One of them is shown in Fig. 2a, the side length of the external corner is relatively long, and the soil slider behind the supporting structure of the external corner can be regarded as a wedge-shaped block passing through the bottom of the foundation pit. The other is shown in Fig. 2b, the side length of the external corner is relatively short, and two adjacent corners constrain the external corner, so the displaced soil can be composed of two parts (zone I and zone II).

**Figure 3:**
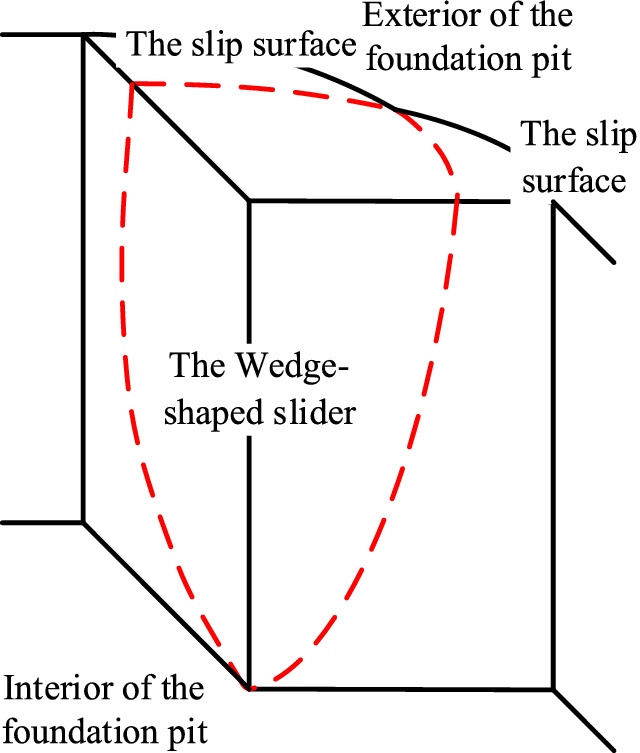
Analysis of failure mechanism for the external corner with long side length.

Assuming that the depth of the foundation pit does not change, only the lengths of the sides on both sides of the external corner change, and the failure mechanisms of the two above-mentioned external corners with equal side lengths are analyzed in conjunction with Figs. 2 and 3:

1. As shown in Fig. 3, when the edge length of the external corner is relatively long, the pit wall will slip in the two directions connected with the external corner of the foundation pit, and the sliding surface will pass through the bottom of the pit. Because of the external corner, the two sliding surfaces will intersect near the external corner of the foundation pit so that the sliders overlap. The overlapping slider is a wedge-shaped block, as shown in Fig. 2a. When the external corner is destroyed, the sliding wedge will slide.
2. As shown in Fig. 2b, considering the interaction between the external corner and the internal corner, the internal corner of the foundation pit can stabilize the deformation of the foundation pit. When the side length of the external corner is relatively small, the soil between the external corner and the internal corner is limited. And the research results of the earth pressure in the limited soil can be used for reference^28–30^. The limited soil on both sides of the external corner will restrict the development of the external corner slip surface so that the slip surface cannot completely intersect with the ground surface. And the displaced soil behind the retaining wall can be composed of the upper area I and the lower area II. In addition, it is worth noting that the failure mode in Fig. 2a applies not only to external corners with equal sides but also to external corners with sufficiently long but unequal sides, while Fig. 2b applies only to external corners with equal sides.

#### Calculation of active earth pressure for the external corner with equal lengths on both sides

**Figure 4:**
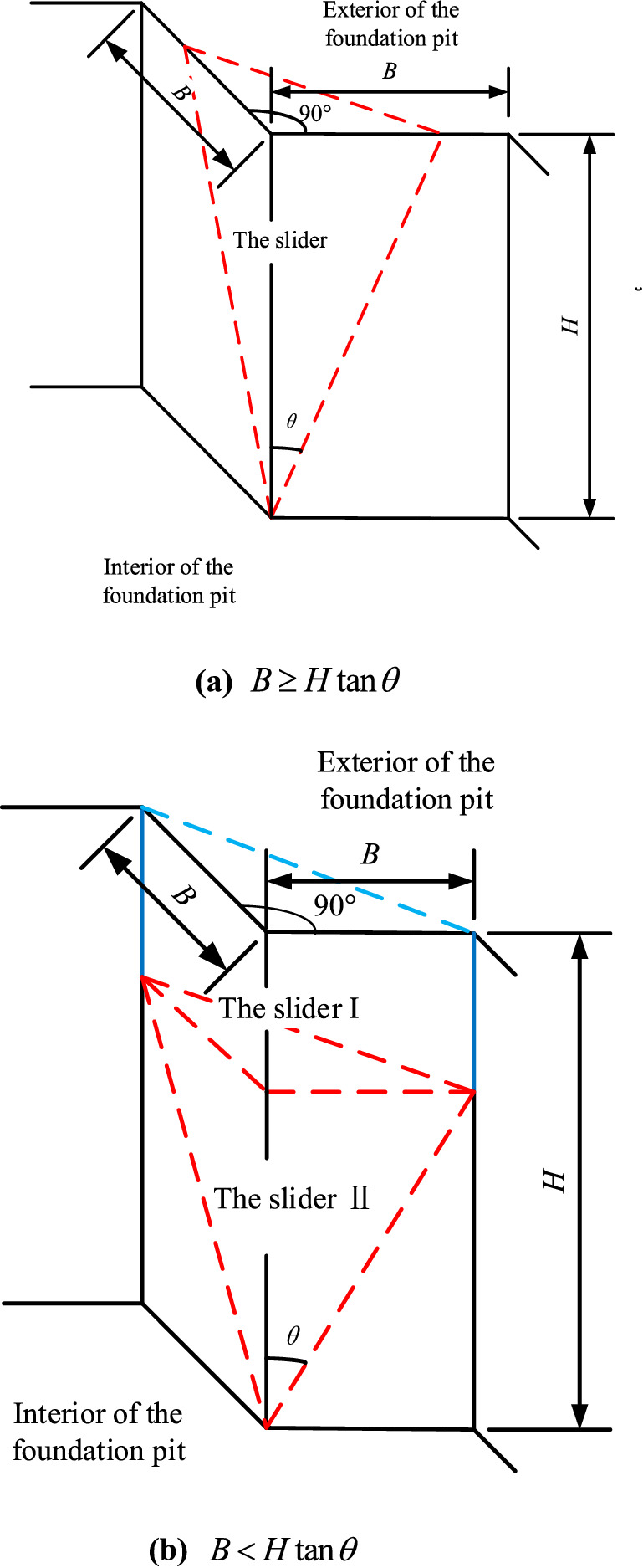
The simplified failure mode of external corners with an angle of 90°.

In excavation engineering, the most common is the external corner with an angle of 90°. Therefore, this paper makes a theoretical analysis of the external corner with an angle of 90°. And taking sand as the research object, cohesion is not considered. To facilitate derivation, when the side length of the external corner with equal side length is relatively long ($B \ge H\tan \theta$), the failure model of the external corner is simplified to the form shown in Fig. 4a. Where *B* is the length of the two sides of the external corner with equal length, *H* is the depth of the foundation pit, *θ* is the angle between the slip surface of the wedge-shaped slider and the vertical line, and the angle of the external corner is 90°. In the same way, when the length of both sides of the equal external corner is relatively short ($B < H\tan \theta$), Fig. 2b is simplified to the form of Fig. 4b.

In deducing the calculation formula of earth pressure, the following basic assumptions are made:

1. The vertical soil stress *σ*_*z*_ is uniformly distributed in the same horizontal plane.
2. The normal stress between the supporting structure and the soil is uniformly distributed at the same height.
3. The sliding surface of the slider passes through the bottom of the foundation pit.
4. The wall is smooth. Therefore, the friction between the supporting structure and the soil is not considered.
5. The displacement mode of the retaining wall is a translational motion mode, so the shear stress between the horizontal microelement layers can be ignored.

**Figure 5:**
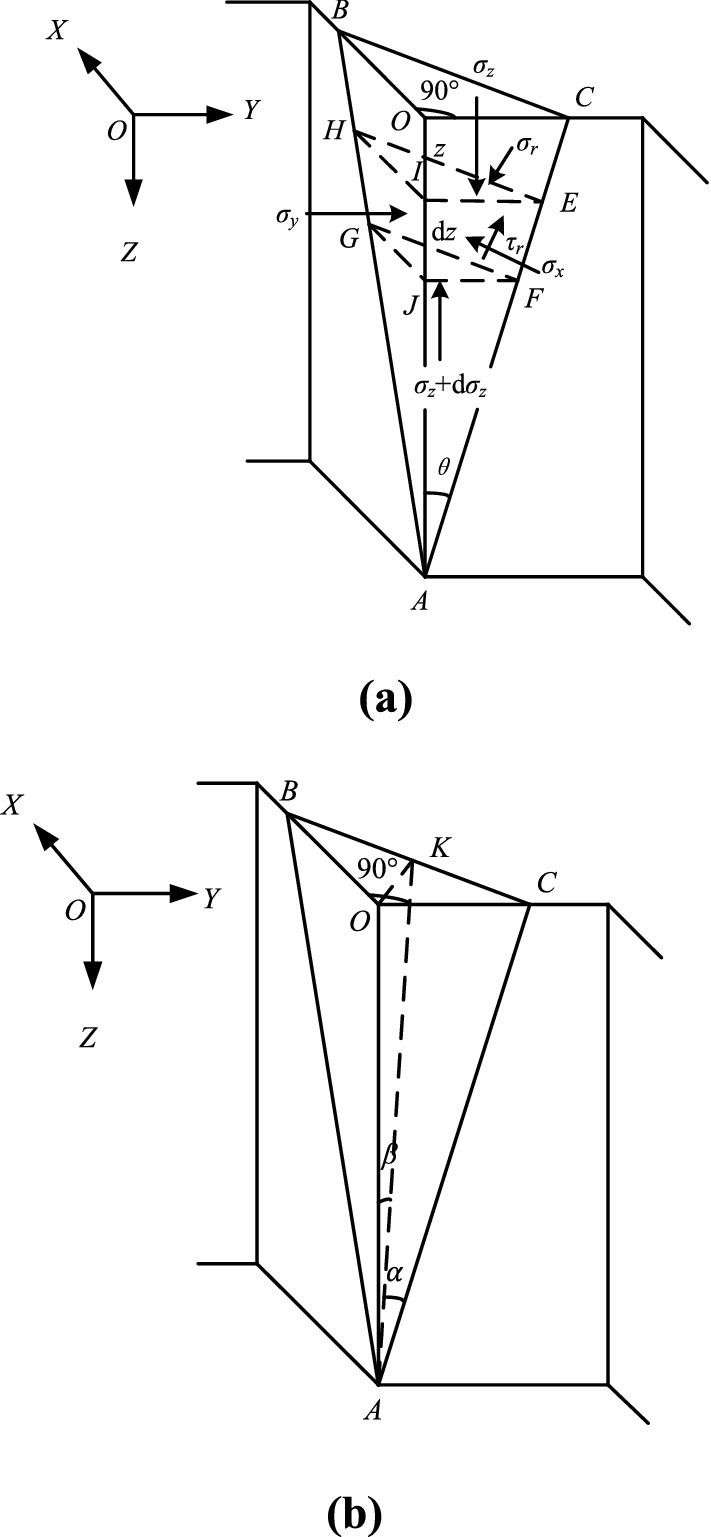
Horizontal microelement stress state of the external corner when $B \ge H\tan \theta$.

When $B \ge H\tan \theta$, as shown in Fig. 5a,b, the earth pressure is theoretically deduced by the horizontal micro-layered limit equilibrium analysis method^16^, and a horizontal microelement with a thickness of dz is taken for analysis. Where *σ*_*r*_ and *τ*_*r*_ are the normal stress and shear stress on the slip plane *AB*C, *x* and *y* are the lengths of *IE* and *HI* on the differential slider, *σ*_*x*_ and *σ*_*y*_ are the normal stresses of the interaction between the wall and soil on the plane *AOC* and *AOB*, *K* is the midpoint of *BC*, *β* (the angle *KAO*) is the angle between the sliding direction of the slider and the vertical line *AO*, *φ* is the angle of internal friction of the soil, and d*W* is the weight of the element.

The static equilibrium differential equation in the vertical direction of the element:

1 $$\sigma_{r} \sin \beta A_{3} + \sigma_{r} \tan \varphi \cos \beta A_{3} + \left( {\sigma_{z} + {\text{d}}\sigma_{z} } \right)A_{4} = {\text{d}}W + \sigma_{z} A_{5}$$

In this equation,

2 $$\left. \begin{aligned} A_{1} & = y{\text{d}}z \hfill \\ A_{2} & = x{\text{d}}z \hfill \\ A_{3} & = \sqrt 2 \cos \alpha \sec \theta x{\text{dz}} \hfill \\ A_{4} & = \frac{{\left( {x - \tan \theta {\text{d}}z} \right)^{2} }}{2} \hfill \\ A_{5} & = \frac{{x^{2} }}{2} \hfill \\ {\text{d}}W & = xy\frac{\gamma }{2}{\text{d}}z \hfill \\ \end{aligned} \right\}$$

where: *A*_1_, *A*_2_, *A*_3_, *A*_4_ and *A*_5_ are the areas of plane *IEFJ*, plane *HIJG*, plane *HEFG*, plane *HEJ* and plane *GJF* respectively.

Since the sides of the external corner are equal, the slider of the external corner should be a symmetrical structure. If its symmetry is considered, there is $\sigma_{x} = \sigma_{y}$ and $x = y$, and then Eq. (2) is substituted into Eq. (1), so Eq. (1) can be simplified as follows:

3 $$\sqrt 2 \sigma_{r} \cos \alpha \sec \theta \left( {\sin \beta + \tan \varphi \cos \beta } \right) + \frac{{{\text{d}}\sigma_{z} }}{{{\text{d}}z}}\frac{x}{2} - \sigma_{z} \tan \theta = x\frac{\gamma }{2}$$

where:

4 $$\left. \begin{aligned} \beta & = \arctan \left( {\frac{\sqrt 2 }{2}\tan \theta } \right) \hfill \\ \alpha & = \arcsin \left( {\frac{\sqrt 2 }{2}\sin \theta } \right) \hfill \\ x& = \left( {H - z} \right)\tan \theta \hfill \\ \end{aligned} \right\}$$

Because the wedge-shaped slider is symmetrical, the equilibrium of the differential slider in both *OB* and *OC* directions is equivalent, and only the static equilibrium equation in one direction is considered, then the static equilibrium differential equation in the *OB* direction is:

5 $$\sigma_{x} A_{1} + \frac{\sqrt 2 }{2}\sigma_{r} A_{3} \tan \varphi \sin \beta = \frac{\sqrt 2 }{2}\sigma_{r} A_{3} \cos \beta$$

Considering the symmetry, *σ*_*x*_ = *σ*_*y*_, *A*_1_ = *A*_2_, *σ*_*x*_ = λ*σ*_*z*_ (λ is the lateral pressure coefficient), and Eq. (2) are substituted into Eq. (5) and then simplified to get:

6 $$\sigma_{x} \frac{1}{{\cos \alpha \sec \theta \left( {\cos \beta - \tan \varphi \sin \beta } \right)}} = \sigma_{r}$$

where the value of λ is calculated by Eq. (7).

7 $$\lambda = \tan^{2} \left( {\frac{\pi }{4} - \frac{\varphi }{2}} \right)$$

Equation (8) can be obtained by substituting Eq. (6) and $x = \left( {H - z} \right)\tan \theta$ into Eq. (3).

8 $$\frac{{A\sigma_{z} }}{H - z} + \frac{{{\text{d}}\sigma_{z} }}{{{\text{d}}z}} = \gamma$$

In this equation,

9 $$A = {2}\sqrt 2 \lambda \frac{\tan \beta + \tan \varphi }{{\tan \theta \left( {1 - \tan \varphi \tan \beta } \right)}} - 2$$

The boundary condition is as follows,

10 $$\left. {\sigma_{z} } \right|_{z = 0} = 0$$

Therefore, the following equation of active earth pressure calculation can be obtained by solving Eq. (8).

11 $$\left. \begin{aligned} \sigma_{z} & = \frac{{\gamma \left( {H - z} \right)\left[ {H^{A - 1} - \left( {H - z} \right)^{A - 1} } \right]}}{{\left( {A - 1} \right)H^{A - 1} }} \hfill \\ \sigma_{x} & = \sigma_{y} = \lambda \sigma_{z} \hfill \\ \end{aligned} \right\}$$

**Figure 6:**
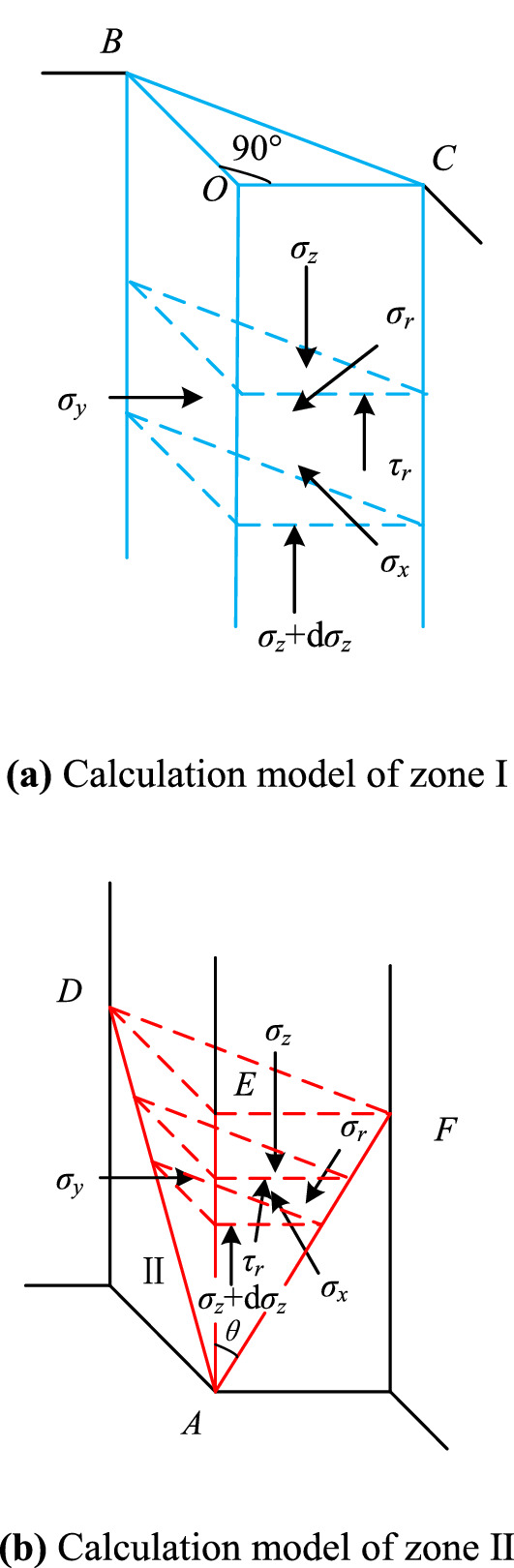
Horizontal microelement stress state of the external corner when $B < H\tan \theta$.

When $B < H\tan \theta$, the slip body is composed of the upper triangular prism (zone I) and the lower wedge (zone II). When the horizontal micro-layered limit equilibrium analysis method is used, the calculation diagram is shown in Fig. 6. The calculation diagram of the upper triangular prism (zone I) is shown in Fig. 6a, and the calculation diagram of the lower wedge (zone II) is shown in Fig. 6b.

1. zone I ($$z \le H - B\cot \theta$$)
  Also, considering the symmetry, the equilibrium equation of the triangular prism in the *OC* direction and the vertical direction is as follows
  12 $$\left. \begin{aligned} \sigma_{r} A_{3} \tan \varphi + \left( {\sigma_{z} + {\text{d}}\sigma_{z} } \right)A_{4} & = {\text{d}}W + \sigma_{z} A_{4} \hfill \\ \sigma_{x} A_{1} + \sigma_{y} A_{2} & = \sqrt 2 \sigma_{r} A_{3} \hfill \\ \end{aligned} \right\}$$
  In this equation,
  13 $$\left. \begin{aligned} A_{1} & = A_{2} = B{\text{d}}z \hfill \\ A_{3} & = \sqrt 2 B{\text{d}}z \hfill \\ A_{4} & = \frac{{B^{2} }}{2} \hfill \\ {\text{d}}W &= \frac{{B^{2} \gamma }}{2}{\text{d}}z \hfill \\ \end{aligned} \right\}$$
  The boundary condition is as follows,
  14 $$\left. {\sigma_{z} } \right|_{z = 0} = 0$$
  where $\sigma_{x} = \lambda \sigma_{z}$, when considering symmetry, $\sigma_{x} = \sigma_{y}$ and $x = y$ is established and combined with Eqs. (12) and (13), the following solutions can be obtained
  15 $$\left. \begin{aligned} \sigma_{z} & = \frac{\gamma }{A ^{\prime} }\left( {1 - e^{ - A ^{\prime} z} } \right) \hfill \\ \sigma_{x} & = \sigma_{y} = \lambda \sigma_{z} \hfill \\ \end{aligned} \right\}$$
  In this equation,
  16 $$A ^{\prime} = \frac{2\sqrt 2 \lambda \tan \varphi }{B}$$
2. zone II ($$z > H - B\cot \theta$$)
  The equilibrium equations of the wedge in the *OC* direction and the vertical direction are as follows,
  17 $$\left. \begin{aligned} \sigma_{r} A_{3} \left( {\sin \beta + \tan \varphi \cos \beta } \right) + \left( {\sigma_{z} + {\text{d}}\sigma_{z} } \right)A_{4} & = {\text{d}}W + \sigma_{z} A_{5} \hfill \\ \sigma_{x} A_{1} + \frac{\sqrt 2 }{2}\sigma_{r} A_{3} \tan \varphi \sin \beta & = \frac{\sqrt 2 }{2}\sigma_{r} A_{3} \cos \beta \hfill \\ \end{aligned} \right\}$$
  In this equation,
  18 $$\left. \begin{aligned} A_{1} & = y{\text{d}}z \hfill \\ A_{2} & = x{\text{d}}z \hfill \\ A_{3} & = \sqrt 2 \cos \alpha \sec \theta x{\text{dz}} \hfill \\ A_{4} &= \frac{{\left( {x - \tan \theta {\text{d}}z} \right)^{2} }}{2} \hfill \\ A_{5} &= \frac{{x^{2} }}{2} \hfill \\ {\text{d}}W &= xy\frac{\gamma }{2}{\text{d}}z \hfill \\ \end{aligned} \right\}$$
  The boundary condition is as follows,
  19 $$\left. {\sigma_{z} } \right|_{z = H - B\cot \theta } = \frac{\gamma }{A ^{\prime} }\left( {1 - e^{ - A ^{\prime} H + A ^{\prime} B\cot \theta } } \right)$$
  where $\sigma_{x} = \lambda \sigma_{z}$, when considering symmetry, $\sigma_{x} = \sigma_{y}$ and $x = y$ is established and combined with Eqs. (17) and (18), the following solutions can be obtained:
  20 $$\left. \begin{aligned} \sigma_{z} & = \frac{{\gamma \left( {H - z} \right)}}{A - 1} + \left( {H - z} \right)^{A} C \hfill \\ \sigma_{x} &= \sigma_{y} = \lambda \sigma_{z} \hfill \\ \end{aligned} \right\}$$
  In this equation,
  21 $$\left. \begin{aligned} A & = 2\sqrt 2 \lambda \frac{\tan \beta + \tan \varphi }{{\tan \theta \left( {1 - \tan \varphi \tan \beta } \right)}} - 2 \hfill \\ C & = \frac{{\gamma \left( {1 - e^{ - A ^{\prime} (H - B\cot \theta )} } \right)\left( {A - 1} \right) - A ^{\prime} \gamma B\cot \theta }}{{A ^{\prime} \left( {A - 1} \right)B^{A} \cot^{A} \theta }} \hfill \\ A ^{\prime} &= \frac{2\sqrt 2 \lambda \tan \varphi }{B} \hfill \\ \end{aligned} \right\}$$
  Therefore, when the side length of the external corner satisfies $B < H\tan \theta$, the calculation equation of the earth pressure around the external corner of the foundation pit is as follows.
  22 $$\sigma_{x} = \left\{ \begin{aligned} & \frac{\lambda \gamma }{{A ^{\prime} }}\left( {1 - e^{ - A ^{\prime} z} } \right),\;\;z \le H - B\cot \theta \hfill \\ & \frac{{\lambda \gamma \left( {H - z} \right)}}{A - 1} + \lambda \left( {H - z} \right)^{A} C,\;\;z > H - B\cot \theta \hfill \\ \end{aligned} \right.$$
  As a result, the equation for calculating the earth pressure of the external corner with equal length on both sides can be obtained when considering the size of different side lengths. When the side length on both sides of the external corner satisfies $B \ge H\tan \theta$, the earth pressure around the external corner is calculated according to Eq. (11). From Eq. (11), it can be seen that the strength of the active earth pressure around the wedge-shaped slider is not affected by the side length *B* of the external corner. The earth pressure strength around the wedge-shaped slider will not change with the side length of the external corner. When the side length of the external corner satisfies the $B < H\tan \theta$, the sliding soil around the external corner is composed of two parts (zone I and zone II). The active earth pressure in zone I ($z \le H - B\cot \theta$) is calculated according to Eq. (15), and the active earth pressure in zone II ($z > H - B\cot \theta$) is calculated according to Eq. (20). The active earth pressure around the external corner will change the side length *B* for the external corner. It is worth noting that Eqs. (11) and (22) can only be applied to soils without cohesion. In cohesive soil, the calculation results will be too conservative if Eqs. (11) and (22) are used to calculate the active earth pressure. Therefore, to improve the accuracy of the calculation results, the cohesion can be calculated as the angle of internal friction^17^.

#### Calculation of active earth pressure resultant force for the external corner in the excavation engineering

When the side length of the external corner satisfies $B \ge H\tan \theta$.

The formula for calculating the resultant force of active earth pressure can be obtained by integrating Eq. (11) in plane *AOC* or *AOB*.

23 $$\begin{aligned} F_{x} &= \iint {\sigma_{x} {\text{d}}x{\text{d}}}z \hfill \\ & = \int_{0}^{H} {\frac{{\lambda \gamma \left( {H - z} \right)^{2} \left[ {H^{A - 1} - \left( {H - z} \right)^{A - 1} } \right]\tan \theta }}{{\left( {A - 1} \right)H^{A - 1} }}{\text{d}}z} \hfill \\ \end{aligned}$$

The following formula for calculating the resultant force of active earth pressure can be obtained from Eq. (23).

24 $$F_{x} = \frac{{\lambda \gamma \tan \theta H^{3} }}{{3\left( {A + 2} \right)}}$$

The resultant action position of active earth pressure in the horizontal x direction is as follows.

25 $$\overline{x} = \frac{{M_{x} }}{{F_{x} }}$$

In this equation,

26 $$\begin{aligned} M_{x} & = \iint\limits_{D} {x\sigma_{x} {\text{d}}x{\text{d}}z} \hfill \\ & = \int_{0}^{H} {\frac{{\lambda \gamma \left( {H - z} \right)^{3} \tan^{2} \theta \left[ {H^{A - 1} - \left( {H - z} \right)^{A - 1} } \right]}}{{2\left( {A - 1} \right)H^{A - 1} }}{\text{dz}}} \hfill \\ & = \frac{{\lambda \gamma \tan^{2} \theta H^{4} }}{{8\left( {A + 3} \right)}} \hfill \\ \end{aligned}$$

Substituting Eq. (26) into Eq. (25), we can get

27 $$\overline{x} = \frac{{M_{x} }}{{F_{x} }} = \frac{3(A + 2)H\tan \theta }{{8(A + 3)}}$$

The resultant action position of active earth pressure in the horizontal z direction is as follows.

28 $$\overline{z} = \frac{{M_{z} }}{{F_{x} }}$$

In this equation,

29 $$\begin{aligned} M_{z} & = \iint\limits_{D} {z\sigma_{x} {\text{d}}x{\text{d}}z} \hfill \\ & = \int_{0}^{H} {\frac{{\lambda \gamma z\left( {H - z} \right)^{2} \left[ {H^{A - 1} - \left( {H - z} \right)^{A - 1} } \right]\tan \theta }}{{\left( {A - 1} \right)H^{A - 1} }}{\text{d}}z} \hfill \\ & = \frac{{\lambda \gamma H^{4} \left( {A + 6} \right)\tan \theta }}{{12\left( {A + 2} \right)\left( {A + 3} \right)}} \hfill \\ \end{aligned}$$

Substituting Eq. (29) into Eq. (28), we can get

30 $$\overline{z} = \frac{{M_{z} }}{{F_{x} }}{ = }\frac{A + 6}{{4A + 12}}H$$

When the side length of the external corner satisfies $B < H\;\tan \theta$.

1. Calculation of resultant force for the active earth pressure in zone I
  Integrating Eq. (15) can get the following formula,
  31 $$\begin{aligned} F_{{x_{1} }} & = \int_{0}^{H - B\cot \theta } {\frac{\lambda \gamma B}{{A ^{\prime} }}\left( {1 - e^{ - A ^{\prime} z} } \right)dz} \hfill \\ & = \frac{{\lambda \gamma B\left( {H - B\cot \theta } \right)}}{A ^{\prime} } + \frac{{\lambda \gamma B\left( {e^{ - A ^{\prime} H + A ^{\prime} B\cot \theta } - 1} \right)}}{{\left( {A ^{\prime} } \right)^{2} }} \hfill \\ \end{aligned}$$
  Since the slider in zone I is a prismatic slider, the action position of the resultant earth pressure in zone I in the x direction is at the midpoint of the side length of the external corner, and the coordinates in the x direction is as follows,
  32 $$\overline{{x_{1} }} = \frac{{M_{{x_{1} }} }}{{F_{{x_{1} }} }} = \frac{B}{2}$$
  The height z of the active earth pressure resultant action point in zone I is as follows,
  33 $$\overline{{z_{1} }} = \frac{{M_{{z_{1} }} }}{{F_{{x_{1} }} }}$$
  In this equation,
  34 $$\begin{aligned} M_{{z_{1} }} & = \int_{0}^{H - B\cot \theta } {\frac{\lambda \gamma B}{{A ^{\prime} }}\left( {1 - e^{ - A ^{\prime} z} } \right)dz} \hfill \\ & = \frac{\lambda \gamma B}{{A ^{\prime} }}\left[ \begin{aligned} & \frac{{\left( {H - B\cot \theta } \right)^{2} }}{2} \\ & + \frac{{\left( {HA ^{\prime} - A ^{\prime} B\cot \theta + 1} \right)e^{{A ^{\prime} \left( {B\cot \theta - H} \right)}} }}{{\left( {A ^{\prime} } \right)^{2} }} - \frac{1}{{\left( {A ^{\prime} } \right)^{2} }} \hfill \\ \end{aligned} \right] \hfill \\ \end{aligned}$$
2. Calculation of resultant force for the active earth pressure in zone II
  Integrating Eq. (20) can get the following formula,
  35 $$\begin{aligned} F_{{x_{2} }} & = \int_{H - B\cot \theta }^{H} {\left[ {\frac{{\lambda \gamma {\text{tan}}\theta \left( {H - z} \right)^{2} }}{A - 1} + \lambda {\text{tan}}\theta \left( {H - z} \right)^{A + 1} C} \right]{\text{d}}z} \hfill \\ & = \frac{{\lambda \gamma B^{3} {\text{cot}}^{2} \theta }}{{3\left( {A - 1} \right)}} + \frac{{\lambda B^{A + 2} {\text{cot}}^{A + 1} \theta }}{A + 2}C \hfill \\ \end{aligned}$$
  The action position of the resultant active earth pressure in the horizontal x direction of zone II is as follows,
  36 $$\overline{x}_{2} = \frac{{M_{{x_{2} }} }}{{F_{{x_{2} }} }}$$
  In this equation,
  37 $$\begin{aligned} M_{{x_{2} }} & = \int_{H - B\cot \theta }^{H} {\left[ \begin{aligned} \frac{{\lambda \gamma \left( {H - z} \right)^{3} \tan^{2} \theta }}{{2\left( {A - 1} \right)}} \hfill \\ + \lambda \frac{{\left( {H - z} \right)^{A + 2} \tan^{2} \theta }}{2}C \hfill \\ \end{aligned} \right]{\text{d}}} z \hfill \\ & = \frac{{\lambda B^{4} \cot^{2} \theta }}{{8\left( {A - 1} \right)}} + \frac{{\lambda B^{A + 3} \cot^{A + 1} \theta }}{{2\left( {A + 3} \right)}}C \hfill \\ \end{aligned}$$
  The height z of the resultant action point of active earth pressure in zone II is as follows,
  38 $$\overline{{z_{2} }} = \frac{{M_{{z_{2} }} }}{{F_{{x_{1} }} }}$$
  In this equation,
  39 $$\begin{aligned} M_{{z_{2} }} & = \int_{H - B\cot \theta }^{H} {\left[ \begin{aligned} \frac{{\lambda \gamma z\left( {H - z} \right)^{2} \tan \theta }}{A - 1} + \hfill \\ z\left( {H - z} \right)^{A + 1} C\tan \theta \hfill \\ \end{aligned} \right]{\text{d}}} z \hfill \\ & = \lambda \gamma B^{3} \cot^{2} \theta \left( {\frac{H}{3A - 3} - \frac{B\cot \theta }{{4A - 4}}} \right) \\ & \quad + \lambda B^{A + 2} C\cot^{A + 1} \theta \left( {\frac{H}{A + 2} - \frac{B\cot \theta }{{A + 3}}} \right) \hfill \\ \end{aligned}$$
  The calculation formula of the total active earth pressure resultant force of zone I and zone II is as follows,
  40 $$F_{x} = F_{{x_{1} }} + F_{{x_{2} }}$$
  The formula for calculating the action point of the total active earth pressure resultant force in Zone I and Zone II is as follows,
  41 $$\left. \begin{aligned} \overline{x} & = \frac{{M_{{x_{1} }} + M_{{x_{2} }} }}{{F_{{x_{1} }} + F_{{x_{2} }} }} \hfill \\ \overline{z} & = \frac{{M_{{z_{1} }} + M_{{z_{2} }} }}{{F_{{x_{1} }} + F_{{x_{2} }} }} \hfill \\ \end{aligned} \right\}$$

## Numerical simulation

### Establishment of finite element model

To further verify the feasibility of the calculation theory and better reflect the magnitude and distribution of active earth pressure at the external corner of the foundation pit. The three-dimensional finite element analysis is carried out in the deep foundation pit by using Midas/GTS software, and there is an external corner with equal lengths on both sides in this foundation pit.

**Figure 7:**
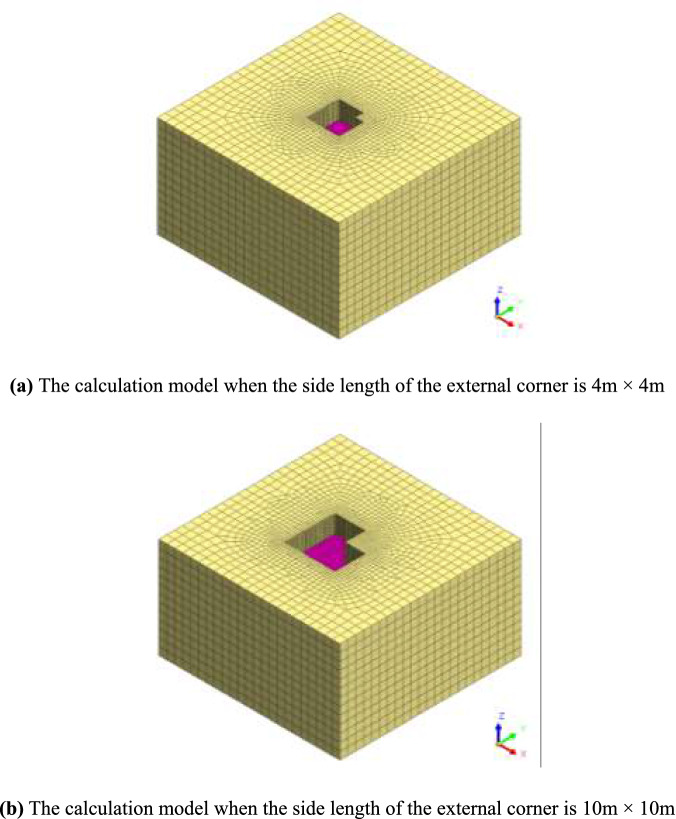
Finite element model in Midas/GTS.

The modified Mohr–Coulomb constitutive model is selected for the soil model. The width affected is 3 to 4 times the depth of excavation and the depth affected is 2 to 4 of the excavation depth^2^. To facilitate the calculation, the soil layer adopts a single sand layer and the cohesion is not considered. The excavation depth of the foundation pit is 9 m, divided into three layers, and each layer is excavated by 3 m. The supporting structure is equivalent to a plate element with a thickness of 0.8 m, embedded depth of 11 m and elastic modulus of 30GPa. The interface element simulates the wall and soil interaction, and the angle of the external corner is 90°. Because the friction between the retaining wall and soil is not considered in the theoretical hypothesis, the roughness factor of the wall-soil interface in the model is 0. The side lengths of the external corner are 3 m × 3 m, 4 m × 4 m, 8 m × 8 m, 10 m × 10 m, and 12 m × 12 m respectively to calculate the model. As shown in Fig. 7, the finite element calculation model of the side length of the external corner is 4 m and 8 m, and the soil parameters are listed in Table 1.

**Table 1:** Parameters of soil.

Soil layer	Unit weight, γ/(kN/m^3^)	Cohesive force, c/kPa	Internal friction angle, φ/(°)	Compression modulus, *E*_a_/MPa
1	17	1	25	43

### Analysis of finite element model

**Figure 8:**
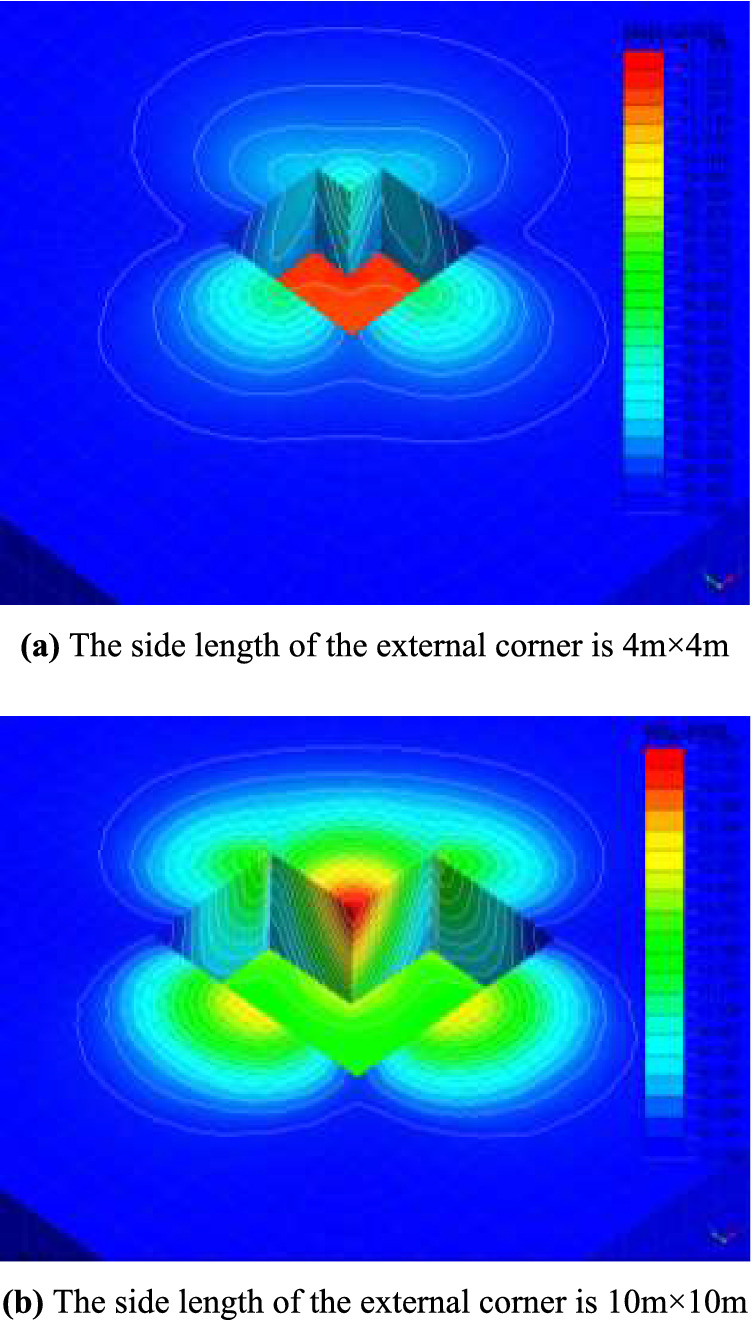
Contour map of displacement contours.

The cloud diagram of the displacement isoline calculated by finite element software is shown in Fig. 8. If the θ is calculated according to ${\pi \mathord{\left/ {\vphantom {\pi 4}} \right. \kern-0pt} 4} - {\varphi \mathord{\left/ {\vphantom {\varphi 2}} \right. \kern-0pt} 2}$, you can get $\theta { = }32.5^\circ$. As shown in Fig. 8a, the side length of the external corner is 4 m and less than $H\tan \theta \approx 5.73{\text{m}}$. It can be seen from Fig. 8a that the displacement of the external corner at this time is consistent with the failure model of the external corner with equal length on both sides proposed in Fig. 2b, both of which can be regarded as composed of the upper prism slider and the lower wedge-shaped slider. It can also be seen from the displacement contour cloud map in Fig. 8b that the soil around the external corner slides as a wedge-shaped slider, which is consistent with the failure model proposed in Fig. 2a.

**Figure 9:**
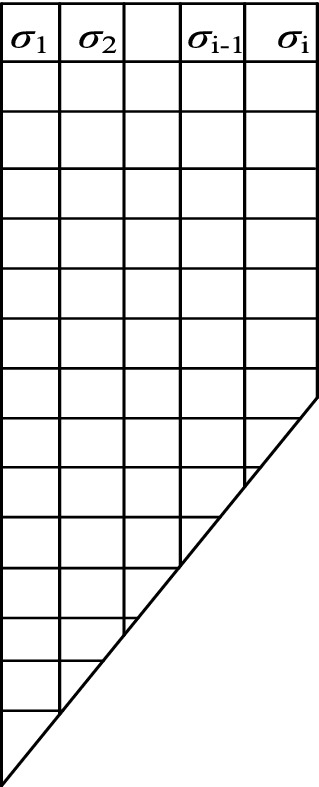
Schematic diagram of partial meshing.

To further prove the feasibility of this method, the simulation value of active earth pressure calculated by finite element software is compared with the calculated value according to this method to verify the rationality of the theory. In the theoretical method of this paper, when deducing the active earth pressure theory of the external corner, the normal stress caused by the interaction between the supporting structure and soil at the same horizontal height is simplified to the uniform distribution. However, in the Midas/GTS finite element software calculation, the active earth pressure obtained by the interface element is not uniformly distributed at the same horizontal height. Therefore, to verify the rationality of the calculation model and theory more reasonably, the variable σ_*v*_ is introduced, as shown in Fig. 9, representing the average earth pressure strength of all monitoring points at the same horizontal height. It is used to quantitatively describe the comprehensive distribution law of earth pressure strength calculated by the software, and the calculation equation is as follows,

42 $$\sigma_{v} = \frac{{\sigma_{1} + \sigma_{2} + \sigma_{3} + \cdots + \sigma_{i - 1} + \sigma_{i} }}{i}$$

where σ_*v*_ is the average value of the active earth pressure strength of all monitoring points at the same horizontal height, σ_*i*_ is the active earth pressure strength of each monitoring point in the same horizontal plane, and *i* is the number of monitoring points.

**Figure 10:**
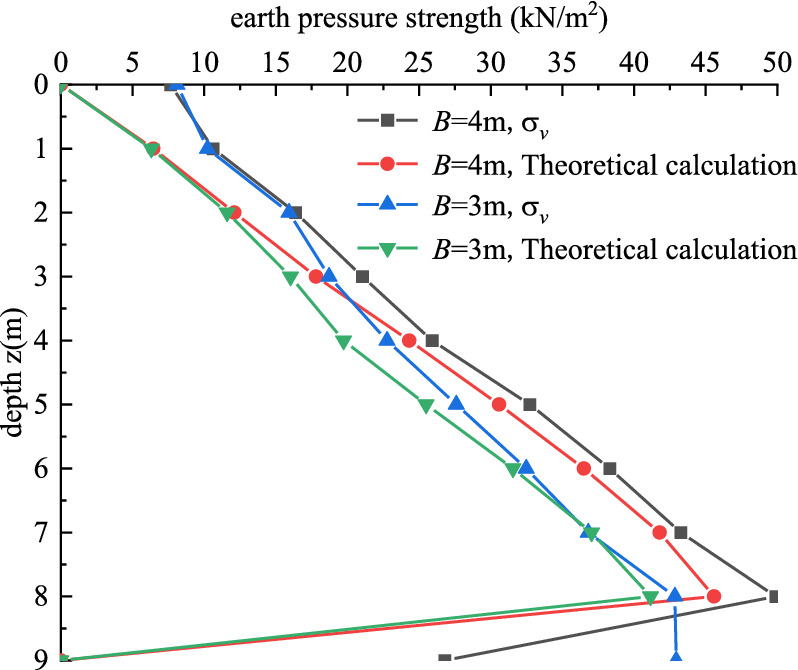
The comparison of calculation results when *B* < *H*tan*θ.*

When the side length of the external corner is 3 m and 4 m respectively ($B < H\tan \theta$), the active earth pressure strength is calculated according to the Eq. (22). Figure 10 shows the comparison between the finite element software simulation value σ_v_ and the theoretical calculation results in this paper when the side length of the external corner is equal to 3 m and 4 m respectively. It can be seen that the earth pressure strength obtained by software simulation and the earth pressure calculated by the theoretical method in this paper are both linearly distributed. The active earth pressure strength calculated by the theoretical method in this paper is slightly smaller than that of the finite element software, but the overall trend of the data is in good agreement with each other. The main reason for the analysis is that when calculating by the finite element software, the soil displacement often can not reach the displacement value required by the active earth pressure in the limit state. Therefore, the active earth pressure strength calculated by the finite element software is slightly larger than that calculated by the theoretical method in this paper. In addition, the theoretical method and software simulation results show that when the lengths of the sides of the external corner are 3 m and 4 m respectively, there is little difference in the active earth pressure strength between them in the range of 2 m from the top of the foundation pit. However, when the depth *z* is greater than 2 m, the two curves appear obvious "bifurcation", which indicates that the earth pressure strength distribution law calculated by the theoretical method in this paper is consistent with the results of the finite element simulation.

**Figure 11:**
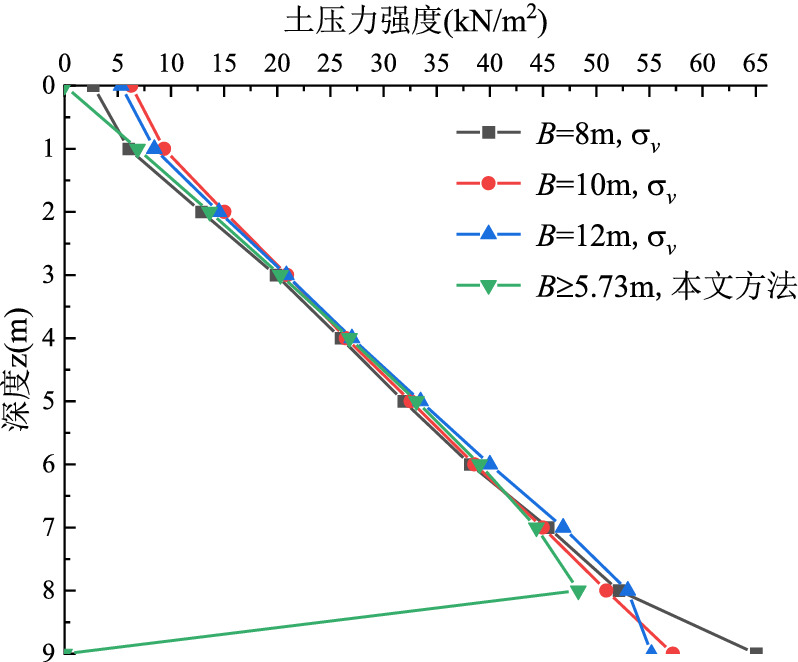
The comparison of calculation results when *B* ≥ *H*tan*θ.*

When the side length of the external corner is 8 m, 10 m and 12 m respectively, the earth pressure strength is calculated according to the Eq. (11). Figure 11 shows the comparison between the simulated value σ_*v*_ and the theoretical calculation results with the side length of 8 m, 10 m and 12 m respectively ($B \ge H\tan \theta$). It can be seen from Eq. (11) that when the side length of the external corner is greater than $H\tan \theta = 5.73m$, the earth pressure strength calculated in this paper will not change with the change of the side length *B*. As shown in Fig. 11, when the side length of the external corner is 8 m, 10 m and 12 m respectively, the curve of σ_*v*_ is almost coincident with the simulation of Midas/GTS software. And it is not much different from the value of earth pressure strength calculated by the theoretical method in this paper. It can be shown that the earth pressure strength calculated by the software does not change with the change of edge length *B*, which is consistent with the theory of this paper. In addition, the earth pressure strength calculated in this paper and the σ_*v*_ obtained by software simulation still have linear distribution.

Figures 10 and 11 show that the earth pressure strength calculated by the theoretical method in this paper is 0 at the bottom of the foundation pit because the method used in this paper is the horizontal micro-layered limit equilibrium analysis method, while the earth pressure strength obtained by this method is usually 0 at the bottom of the foundation pit. In addition, when the depth z is equal to 9 m, the monitoring point is located in the corner of the bottom of the foundation pit, which is affected by various factors such as stress concentration, so the stress distribution at this point is more complex, so as shown in Figs. 10 and 11, the finite element simulation value σ_*v*_ at the bottom of foundation pit shows the irregular distribution.

## Conclusion

In this paper, the failure model of the external corner with equal side length on both sides is established in excavation engineering . The calculation formula of active earth pressure for this type of external corner under different side length is derived according to the established failure model. The main conclusions are as follows:

1. According to the difference in the side length, the external corner can be divided into two categories. The first type is that the length of the side length *B* is greater than $H\tan \theta$, and the second is that the side length is less than $H\tan \theta$. When the first type of external corner is damaged, it can be considered the sliding failure of the wedge-shaped slider. When the second type of external corner is damaged, the soil slider can be composed of two parts.
2. When the turning angle of the external corner is 90°, the active earth pressure around the two kinds of the external corner is deduced theoretically by using the horizontal micro-layered limit equilibrium analysis method. The formulas for calculating the strength of active earth pressure and the resultant force and acting point of earth pressure are obtained respectively. For the first kind of the external corner, the active earth pressure in the range of the external corner wedge-shaped slider will not change with the side length of the external corner, while for the second kind of the external corner, the earth pressure in the range of the external corner slider will change with the change of the side length for the external corner.
3. In this paper, the theoretical calculation results are compared with the simulation results of Midas/GTS software. The comparison results show that there is little difference between the earth pressure values calculated by the theoretical method and the simulation values of Midas/GTS software, and the magnitude and distribution of the main earth pressure values are the same, which proves that the method in this paper is feasible and reasonable.

## Data Availability

All data generated or analyzed during this study are included in this published article.

## References

[1] Di, W. Monitoring and analysis of deformation of a foundation pit in Lanzhou. In *Proceedings of the 5th International Conference on Green Materials and Environmental Engineering, IOP Conference Series-Earth and Environmental Science* vol. 453, 124–130 (2019).

[2] Ou CY, Chiou DC, Wu TS (1996). Three-dimensional finite element analysis of deep excavations. J. Geotech. Eng..

[3] Ou CY, Shiau BY (1998). Analysis of the corner effect on excavation behaviors. Can. Geotech. J..

[4] Ou CY, Shiau BY, Wang IW (2000). Three-dimensional deformation behavior of the Taipei National Enterprise Center (TNEC) excavation case history. Can. Geotech. J..

[5] Ou CY, Hsieh PG, Lin YL (2010). Performance of excavations with cross walls. J. Geotech. Geoenviron. Eng..

[6] Finno RJ, Blackburn JT, Roboski JF (2007). Three-dimensional effects for supported excavations in clay. J. Geotech. Geoenviron. Eng..

[7] Li Y (2022). Analysis of corner effect of diaphragm wall of special-shaped foundation pit in complex stratum. Front. Earth Sci..

[8] Ding Z, Jin J, Han TC (2018). Analysis of the zoning excavation monitoring data of a narrow and deep foundation pit in a soft soil area. J. Geophys. Eng..

[9] Tan, J., Zheng, X., Sun, Y., Shao, G. & Chen, Y. Analysis of pit corner effect of special-shaped foundation pit of subway station. In *2nd International Conference on Oil & Gas Engineering and Geological Sciences, IOP Conference Series: Earth and Environmental Science* vol. 558, 032032. 10.1088/1755-1315/558/3/032032 (2020).

[10] Liu W, Li T, Wan J (2022). Deformation characteristic of a supported deep excavation system: A case study in red sandstone stratum. Appl. Sci..

[11] Tan Y, Wei B, Diao Y, Zhou X (2014). Spatial corner effects of long and narrow multipropped deep excavations in Shanghai soft clay. J. Perform. Constr. Facil.

[12] Hefny A, Al-Atroush ME, Abualkhair M, Alnuaimi MJ (2020). Three-dimensional response of the supported-deep excavation system: Case study of a large scale underground metro station. Geosciences.

[13] Xiao H, Zhou S, Sun Y (2019). Wall deflection and ground surface settlement due to excavation width and foundation pit classification. KSCE J. Civ. Eng..

[14] Li DP, Tang DG, Yan FG, Huang M (2014). Mechanics of deep excavation’s spatial effect and soil pressure calculation method considering its influence. J. Zhejiang Univ. Eng. Sci..

[15] Lin QT, Zhu JM, Kang Y (2015). Active spatial earth pressure behind retaining wall considering arching effects of soil. Chin. J. Rock Mech. Eng..

[16] Gu WC (2005). Calculation Manual for Earth Pressure on Retaining Wall.

[17] Wang P (2021). A theoretical study on the spatial effect of water-rich foundation pit instability failure. AIP Adv..

[18] Shi F, Lu KL, Yin ZK (2021). Determination of three-dimensional passive slip surface of rigid retaining walls in translational failure mode and calculation of earth pressures. Rock Soil Mech..

[19] Yang XQ, Liu ZD, He SX (1998). Research about spatial effect of deep pit supporting. Chin. J. Geotech. Eng..

[20] Li D, Zhang QC, Jin G, Wang L (2015). Analytical solution of earth pressure on supporting structure of deep foundation pit considering arching effects. Rock Soil Mech..

[21] Zhao W, Chen C, Li S, Pang Y (2014). Researches on the influence on neighboring buildings by concave and convex location effect of excavations in soft soil area. J. Intell. Robot. Syst..

[22] Zhang M, Wang X, Yang GC, Wang Y (2011). Numerical investigation of the convex effect on the behavior of crossing excavations. J. Zhejiang. Univ-Sci. A..

[23] Moradi M, Pooresmaeili BA, Sabermahani M (2020). Effect of nail arrangement on the behavior of convex corner soil-nailed walls. J. Geotech. Geoenviron. Eng..

[24] Dou HQ, Wang H, Wu FB, Xi RS (2018). Corner effects of deep excavations with multi exposed corners in square crossing of utility tunnel. J. Eng. Geol..

[25] Pan H, Zhou CF, Cao H (2008). Analysis of spatial effect and deformation of corner of composite soil nailing walls. Rock Soil Mech..

[26] Wang F, Guo HX (2006). Three-dimensional numerical analysis of spatial effect of foundation pit with yang angle. Railw. Eng..

[27] Wu ZM, Tu YM (2007). Space effect of soil-nailing excavation protection. Rock Soil Mech..

[28] Lai F, Zhang N, Liu S, Yang D (2022). A generalised analytical framework for active earth pressure on retaining walls with narrow soil. Geotechnique.

[29] Lai F, Yang D, Liu S, Zhang H, Cheng Y (2022). Towards an improved analytical framework to estimate active earth pressure in narrow c–ϕ soils behind rotating walls about the base. Comput. Geotech..

[30] Yang D, Lai F, Liu S (2022). Earth pressure in narrow cohesive-fictional soils behind retaining walls rotated about the top: An analytical approach. Comput. Geotech..

